# Molecular and cellular factors control signal transduction via switchable allosteric modulator proteins (SAMPs)

**DOI:** 10.1186/s12918-016-0274-3

**Published:** 2016-04-27

**Authors:** Heiko Babel, Ilka B. Bischofs

**Affiliations:** Center for Molecular Biology (ZMBH), University of Heidelberg, Heidelberg, Germany; Center for the Quantitative Analysis of Molecular and Cellular Biosystems (BioQuant), University of Heidelberg, Heidelberg, Germany

**Keywords:** Two-Component signal transduction, Modulator proteins, Quorum sensing, Allostery, Kinetic model, Rap-Phr-Systems

## Abstract

**Background:**

Rap proteins from *Bacilli* directly target response regulators of bacterial two-component systems and modulate their activity. Their effects are controlled by binding of signaling peptides to an allosteric site. Hence Raps exemplify a class of monomeric signaling receptors, which we call *switchable allosteric modulator proteins* (SAMPs). These proteins have potential applications in diverse biomedical and biotechnical settings, but a quantitative understanding of the impact of molecular and cellular factors on signal transduction is lacking. Here we introduce mathematical models that elucidate how signals are propagated though the network upon receptor stimulation and control the level of active response regulator.

**Results:**

Based on a systematic parameter analysis of the models, we show that key features of the dose-response behavior at steady state are controlled either by the molecular properties of the modulator or the signaling context. In particular, we find that the biochemical activity (i.e. non-enzymatic vs. enzymatic) and allosteric properties of the modulator control the response amplitude. The Hill coefficient and the EC_50_ are controlled in addition by the relative ligand affinities. By tuning receptor properties, either graded or more switch-like (memory-less) response functions can be fashioned. Furthermore, we show that other contextual factors (e.g. relative concentrations of network components and kinase activity) have a substantial impact on the response, and we predict that there exists a modulator concentration which is optimal for response amplitude.

**Conclusion:**

We discuss data on Rap-Phr systems in *B. subtilis* to show how our models can contribute to an integrated view of SAMP signaling by combining biochemical, structural and physiological insights. Our results also suggest that SAMPs could be evolved or engineered to implement diverse response behaviors. However—without additional regulatory controls—they can generate rather variable cellular outputs.

**Electronic supplementary material:**

The online version of this article (doi:10.1186/s12918-016-0274-3) contains supplementary material, which is available to authorized users.

## Background

For almost all forms of life on Earth, the ability to process environmental information is vital for adaptation and survival. For cellular organisms such as bacteria, this function is carried out by biochemical signaling systems. Two-component systems, each consisting of a sensor histidine kinase and a response regulator, are a common form of bacterial signal transduction. The histidine kinase responds to the presence of specific signals that modulate its auto-phosphorylation activity and transfers the phosphoryl group to the response regulator, which in most cases activates or represses gene expression in accordance with its phosphorylation status [1]. In addition to this basic signaling machinery, nature has come up with proteins that target either the histidine kinase or the response regulator directly, and thereby modulate the output. In principle such a modulator protein could interfere with any step in the signal transduction process, and indeed the various modulator proteins that have been identified in bacteria make use of a wide variety of control mechanisms (reviewed in [2]).

The Rap proteins of *Bacillus* are one of the best characterized classes of modulator proteins, and are encoded not only in the chromosomal genome [3] but also on mobile genetic elements including plasmids [4–7], integrated conjugative elements (ICE) [8] and temperate phages [9]. They have been best studied in the model organism *Bacillus subtilis.* Here, Raps interfere with three central signaling systems [10–12] that control important adaptive phenotypes such as the initiation of sporulation, exofactor synthesis, swarming motility, biofilm formation and horizontal gene transfer [13]. Some Raps, including the first ones to be biochemically characterized [10], act as enzymes that form a stable complex with the activated (intermediate) response regulator of the sporulation pathway and promote its dephosphorylation [9, 14]. Indeed, these enzymes are responsible for the family’s name: *R*esponse-regulator *a*spartate *p*hosphatases. However, the name is misleading, since not all Raps act as phosphatases. Other Raps have been found to utilize a non-enzymatic form of modulatory signaling: they bind to the response regulator (regardless of its phosphorylation status [11, 15]) and inhibit the ability of the transcription factor to bind to DNA [11, 12]. Finally a Rap can act as bi-functional modulator protein and interact with two signaling systems by employing the two alternative modes of enzymatic and non-enzymatic response-regulator inhibition [16].

Raps are a particularly interesting group of modulator proteins because their activity is “switchable” through the action of signaling molecules. Most *rap* genes are found in a gene cassette together with a small open reading frame called *phr* (for: *ph*osphatase *r*epressor). *phrs* code for small peptides that are exported from the cell, undergo peptide processing and are then re-imported as mature signaling peptides into the cell, where they interact with their cognate Raps and inhibit their activity towards the target response regulator [3, 17]. In rare instances a cognate *phr* gene is missing or has undergone mutations that render it inactive against the modulator Rap protein [18]. Although the biological function of Phr signaling peptides is not entirely clear and often controversially debated [17], experimentally (at least some) Phrs can be transmitted from cell-to-cell and could therefore serve in intercellular communication [3, 19]. Theoretical models also show that such systems are capable of encoding information about cell densities in the concentration profile of signals, thereby supporting a role for these peptides as quorum-sensing molecules [20]. Therefore Raps are frequently referred to as quorum-sensing receptors in the literature. Since Phrs typically counteract the activity of the modulator they function as inverse agonists.

Recently tremendous progress has been made in elucidating the structural basis of modulator-protein-based signal transduction involving Rap proteins and Phr signaling peptides [21–24]. These studies indicate that the Phr signaling peptide and the response regulator bind to the modulator protein at non-overlapping sites. Hence, the two ligands do not compete for a common binding site on the modulator protein. Instead, binding of the signal causes a structural change in the modulator which modifies its ability to interact with the response regulator at a distant site. Thus, Phr signaling occurs via an allosteric mechanism and hence Rap proteins may be termed *switchable allosteric modulator proteins* (SAMPs). Like class A G-protein-coupled receptors in eukaryotes, which function as allosteric monomers [25], Rap receptors have also been found to be monomers in solution (although some may be dimers) and they interact with both the Phr signal and with the response regulator in a 1:1 stoichiometry [11, 14, 21–24, 26]. Hence, there is only a single allosteric binding site for each ligand on the Rap receptor. This distinguishes Rap receptors from a broad class of allosteric protein receptors which act as oligomers (reviewed in [27]).

Allostery is the primary mode used for cellular signal transduction in nature. From a theoretical point of view, mathematical models, such as the classical Monod-Wyman-Changeux model for oligomeric receptors (recently reviewed in [27]) or the ternary complex model used to describe G-protein-coupled receptors (recently reviewed in [28]), laid the foundation that enabled a better understanding of allosteric signal transduction through cellular networks [29] and facilitated the development of biomedical applications [28, 30]. SAMPs, however, in spite of their important function in controlling bacterial signal transduction, the large amount of experimental work that has been done on Rap receptors in the recent past, and their diverse potential applications [21–24], have received relatively little attention from the modelling community [31–33].

**Fig. 1:**
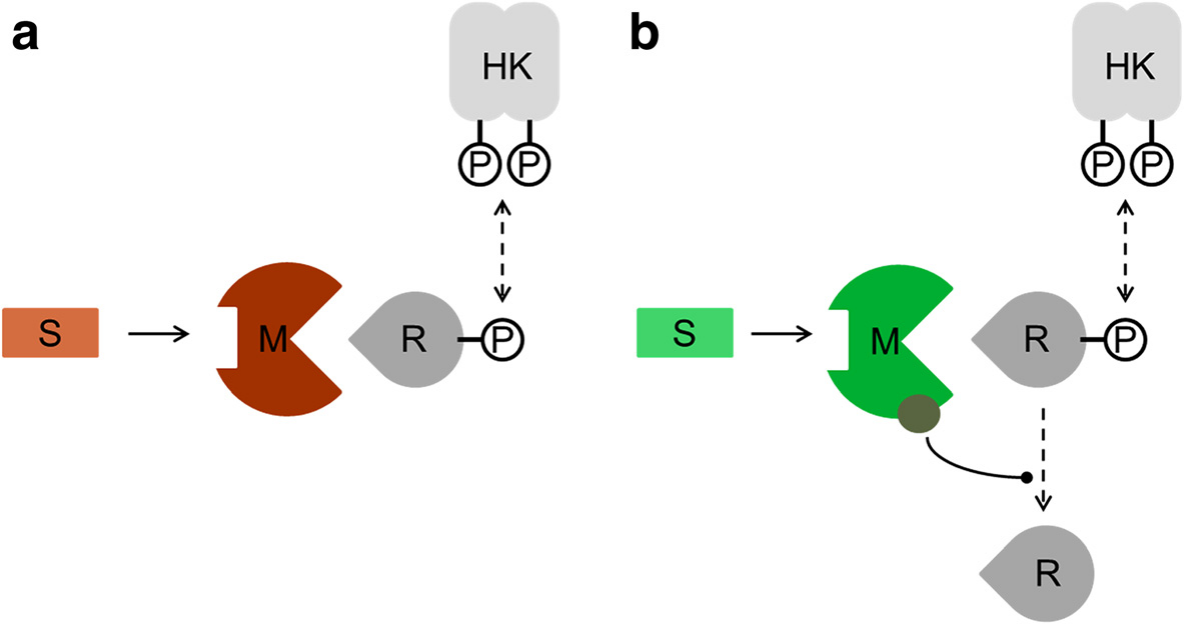
Modes of action of bacterial signaling networks that are controlled by switchable allosteric modulator proteins (SAMPs). SAMPs target bacterial two-component signal transduction systems (grey) by interacting with response regulators. The generic two-component signal transduction system consists of a histidine kinase (HK) and the response regulator (R) phosphorylated by it. The modulator protein (M) targets R and its action on R is allosterically controlled by the signal S. There are two classes of SAMPs. **a** Binding modulators (red) act non-enzymatically and simply sequester R. **b** Enzymatic modulators (green) contain a functional site (grey circle), bind to the R and deactivate it by promoting its dephosphorylation

We set out to fill this gap by developing mathematical models to investigate quantitatively how SAMPs function as allosteric receptors and propagate signals through the cellular signaling network to control the activity of the response regulator. To this end, we differentiate between two types of SAMPs based on their biochemical properties. The first comprises modulator proteins which act by sequestering the response regulator, and the second encompasses modulator proteins that act enzymatically and deactivate the response regulator. We refer to the former as *binding modulators* (Fig. 1a) and to the latter as *enzymatic modulators* (Fig. 1b), respectively.

We then use our models to investigate how the biochemical and allosteric properties of the signaling receptor affect the steady-state response of the signaling network when SAMPs receive a signal. As a qualitative read-out for the response characteristics we determine whether the signal acts as an agonist or an inverse agonist. As a quantitative read-out for response behavior we focus on the dynamic output range (i.e. the maximal response amplitude), the Hill coefficient and the *EC*_*50*_ of the resulting dose-response curves. Moreover, the overall signaling response is expected to depend not only on the molecular properties of the receptor, but also on the “signaling context”, i.e. the “state” of the signaling network—which is largely determined by the concentrations of the network components and the overall level of activity in the two-component system. Hence we also study how the signaling context shapes response behavior and seek to define conditions that optimize information transmission from the receptor through the cellular network to the response regulator. By conducting a systematic parameter analysis of our models, we arrived at quantitative predictions which could provide deeper insight into the functions of natural SAMPs and facilitate the development of applications in the future.

## Models

### The binding modulator model

SAMPs modulate bacterial signal transduction by regulating the activity of a response regulator. The most basic form to modulate signal transduction is to sequester the response regulator. This mode of regulation could, in principle, function without a phosphorylation-dephosphorylation cycle. In fact the majority of bacterial signaling systems are one-component systems where a signal directly controls a response regulator without phosphorylation [34]. As the targets of many Raps are still unknown, it is conceivable that some Raps interact with the response regulators of a one-component system. Notably, Raps in *Bacillus subtilis* which target the response regulators of a two component system do not distinguish between the phosphorylation status of the response regulator and bind to both molecular species equally well [11, 15]. This suggests that in this case the relevant output of SAMP-mediated signaling is the total amount of “free” response regulator in the cell, i.e. the sum of un-sequestered phosphorylated and un-phosphorylated response regulator. Thus, for modeling the action of a binding modulator we will focus on its sequestering action and neglect the phosphorylation status of the response regulator.

**Fig. 2:**
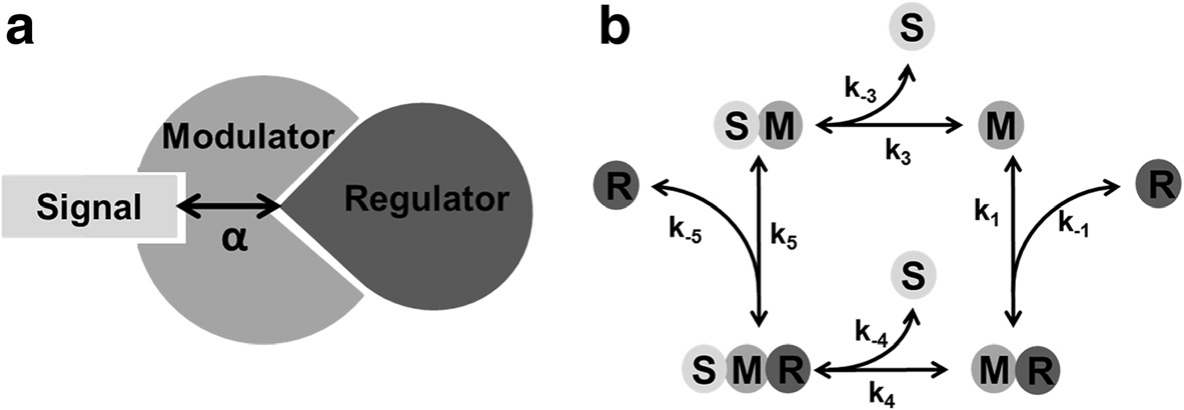
Allosteric switching of a binding modulator. **a** Signal and response regulator bind to a modulator protein at distinct effector sites and influence each other allosterically. The cooperativity *α* quantifies the magnitude and sign of the cooperative effect between R and S. **b** Biochemical reaction scheme depicting the molecular complexes and reactions considered in the binding modulator model. M: modulator, R: response regulator, S: signal

In a binding modulator system the modulator binds to the response regulator and effectively reduces the concentration of free response regulator available. Any signaling factor that binds to the modulator at an allosteric site may alter its conformation and thus modify its ability to bind and sequester the response regulator. On the other hand, binding of the response regulator to its binding site may itself have an allosteric effect, and act as an allosteric regulator for the signal, thereby governing the ability of the *signal* to bind to the modulator. This mutual allosteric interaction can occur in two directions. In cases of “positive cooperativity”, binding of the signal increases the affinity of the modulator for the response regulator and vice versa. In systems with “negative cooperativity”, interaction of the modulator with one ligand diminishes its affinity for the other (Fig. 2a).

Figure 2b shows the corresponding signaling network in form of a biochemical reaction network. The modulator *M* can bind to the signal *S* to form the signal-modulator complex *SM*, and to the regulator *R* to form the complex *MR*. The reaction scheme can be modelled with the help of ordinary differential equations.

#### Kinetic model

We assume mass-action kinetics to describe the dynamics of the concentration of the reactants [*X*]. This gives rise to the following set of ordinary differential equations:

1a $$ \frac{d\left[MR\right]}{dt}=-\left({k}_{\mathit{\hbox{-}} 1}+{k}_4\left[S\right]\right)*\left[MR\right]+{k}_1\left[R\right]\left[M\right]+{k}_{\mathit{\hbox{-}} 4}\left[SMR\right] $$

1b $$ \frac{d\left[SMR\right]}{dt}=-\left({k}_{\mathit{\hbox{-}} 5}+{k}_{\mathit{\hbox{-}} 4}\right)\left[SMR\right]+{k}_5\left[SM\right]\left[R\right]+{k}_4\left[S\right]\left[MR\right] $$

1c $$ \frac{d\left[SM\right]}{dt}=-\left({k}_{\mathit{\hbox{-}} 3}+{k}_5\left[R\right]\right)\left[SM\right]+{k}_3\left[S\right]\left[M\right]+{k}_{\mathit{\hbox{-}} 5}\left[SMR\right] $$

Since this system describes a closed set of reversible reactions, at thermodynamic equilibrium the dissociation constants *K*_*i*_ *= k*_*−i*_*/k*_*i*_ must obey the principle of detailed balance, which gives rise to the following relationship [35]:

2 $$ \frac{K_4}{K_3}=\frac{K_5}{K_1} $$

Protein binding events in prokaryotes occur on timescales of seconds to a few minutes and are thus considerably faster than the typical timescale of fluctuations in protein and signaling peptide concentrations [11, 36]. We therefore assume that the total concentrations of the response regulator [*R*_*T*_], the modulator [*M*_*T*_] and the signal [*S*_*T*_] are constant, which leads to the following mass-balance equations:

3a $$ \left[{R}_T\right]=\left[R\right]+\left[MR\right]+\left[SMR\right] $$

3b $$ \left[{S}_T\right]=\left[S\right]+\left[SM\right]+\left[SMR\right] $$

3c $$ \left[{M}_T\right]=\left[M\right]+\left[SM\right]+\left[MR\right]+\left[SMR\right] $$

We note that the simple binding modulator model is structurally identical to the classical ternary complex model that is used to describe allosteric G-protein coupled receptors [37]. However, the relevant signaling output is different for the two models. In bacterial signaling systems the behavior of the cell is controlled by the concentration of the free response regulator. We therefore use the steady-state concentration of the free regulator as a function of the signal strength, i.e. [*R*] ([*S*_*T*_]), to describe the dose-response behavior of the cellular network upon stimulation of the modulator.

#### Parameter classification

To quantitatively analyze the system response upon receptor stimulation it is useful to classify the model’s parameters into two categories. Class I comprises parameters that describe the molecular properties of the modulator, while Class II comprises all other parameters which determine the “signaling context” or the “state” of the signaling system, such as the total concentrations of regulator [*R*_*T*_] and modulator [*M*_*T*_]. Class II parameters may be actively controlled by the cell by altering gene expression depending on environmental conditions, and they might also show considerable variability from cell to cell—due to gene expression noise.

#### Effective class I control parameters

The most important Class I parameter is α as defined in Eq. (2):

4a $$ \alpha =\frac{K_4}{K_3}=\frac{K_5}{K_1} $$

One can use *α* to quantify the sign and the magnitude of cooperativity in the binding reactions of the signal and the response regulator to the modulator [37]. For positive cooperativity (*α* < 1) binding of the signal increases the affinity of the modulator for the regulator and vice versa. On the other hand, if the cooperativity is negative (*α* > 1) binding of the signal inhibits the binding of the regulator and vice versa. In addition, we may define a second Class I parameter:

4b $$ \beta =\frac{K_3}{K_1}=\frac{K_4}{K_5} $$

*β* relates the affinity of the modulator for the signal to the affinity of the modulator for the response regulator (in the absence of the other ligand). If *β* >1, the modulator has a higher affinity for the response regulator than for the signal, and if *β* < 1 the signal binds with a higher affinity to the modulator than the regulator does.

#### Effective class II control parameters

A key Class II parameter is given by the relative modulator concentration defined as:

4c $$ \mu =\frac{\left[{M}_T\right]}{\left[{R}_T\right]} $$

Finally, the Class I and Class II parameters introduced above are evaluated relative to the absolute dissociation constant *K*_*1*_, which describes the interaction between the response regulator and the modulator and the absolute regulator concentration [*R*_*T*_]:

4d $$ {K}_{R 1}=\frac{K_1}{\left[{R}_T\right]} $$

### The enzymatic modulator model

**Fig. 3:**
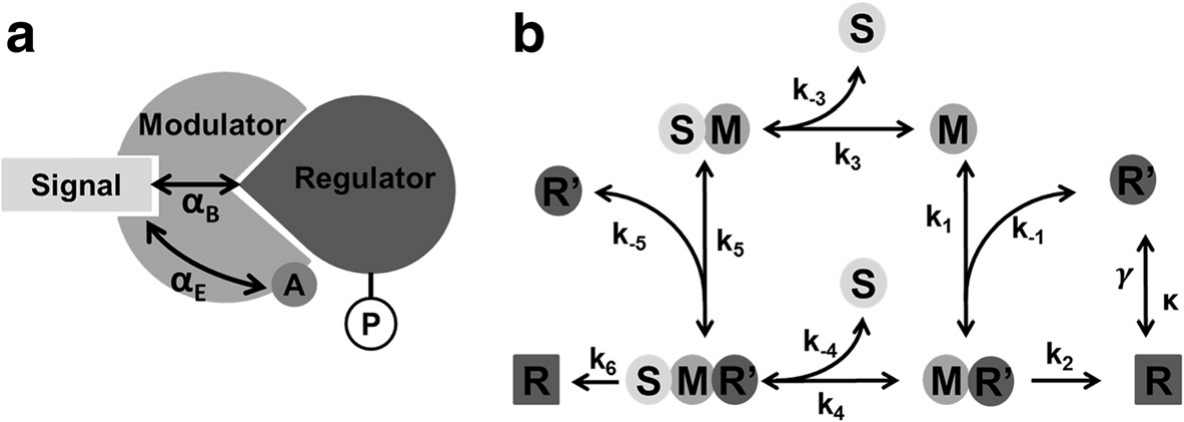
Allosteric switching of an enzymatic modulator. **a** The signal and the activated response regulator bind at distinct effector sites to an enzymatic modulator. “A” denotes the active site. Enzymatic modulators have in principle two allosteric interaction modes: *α*_*B*_ denotes the allosteric effect on ligand binding and *α*_*E*_ that on the enzymatic reaction, respectively. **b** Biochemical reaction scheme depicting the molecular complexes and reactions considered in the enzymatic modulator model. M: modulator, R: inactive response regulator, R’: active response regulator, S: signal

Like any binding modulator, an enzymatic modulator first binds the active regulator and enzymatically inactivates it in a second, separate step. In this case, there are two allosteric mechanisms that derive from the molecular properties of the modulator. As with the binding modulator there is a mutual allosteric influence of the binding affinities. In addition, the enzymatic activity of the modulator may be subject to allosteric regulation, i.e. the signal and the regulator can in principle stimulate or inhibit the enzymatic activity. As a result, the allosteric properties of the modulator protein may be described by two parameters, one describing the sign and strength of binding cooperativity and the other capturing the sign and strength of enzymatic cooperativity (Fig. 3a).

Figure 3b shows the corresponding signaling network for an enzymatic modulator system. We assume that the regulator is subject to a covalent modification cycle [38] and is intrinsically deactivated with rate *γ* [39]. We therefore introduce phosphotransfer reactions into our model by adding a linear activation term with rate *κ* and an inactivation term with rate *γ*, which denotes the effective dephosphorylation rate due to intrinsic phosphatase activity and/or interaction with a bi-functional kinase in the linear regime [38, 40]. Moreover, the active regulator *R’* can be deactivated at different rates, *k*_*2*_ or *k*_*6*_, by binding to the modulator or the modulator/signal complex respectively. The core network that describes the interactions of the modulator with its interaction partners is analogous to the binding modulator model. The active regulator *R’* and the signal *S* bind allosterically to the enzymatic modulator *M*. The signal *S* and the active regulator *R* can form the complexes *SM* and *MR’*, respectively. These complexes in turn can form the ternary complex *SMR’*. We note that for enzymatic modulators from the Rap family it has been shown experimentally that the modulator forms a stable complex with the activated response regulator [14]. This feature was taken into account in setting up the architecture of our model.

#### Kinetic model

Following similar reasoning to before, we describe the enzymatic modulator system by the following ordinary differential equations:

5a $$ \frac{d\left[MR\hbox{'}\right]}{dt}=-\left({k}_{- 1}+{k}_2+{k}_4\left[S\right]\right)*\left[MR\hbox{'}\right]+{k}_1\left[R\hbox{'}\right]\left[M\right]+{k}_{\mathit{\hbox{-}} 4}\left[SMR\hbox{'}\right] $$

5b $$ \frac{d\left[SMR\hbox{'}\right]}{dt}=-\left({k}_{- 5}+{k}_{- 4}+{k}_6\right)\left[SMR\hbox{'}\right]+{k}_5\left[SM\right]\left[R\hbox{'}\right]+{k}_4\left[S\right]\left[MR\hbox{'}\right] $$

5c $$ \frac{d\left[SM\right]}{dt}=-\left({k}_{- 3}+{k}_5\left[R\hbox{'}\right]\right)\left[SM\right]+{k}_3\left[S\right]\left[M\right]+{k}_{- 5}\left[SMR\hbox{'}\right] $$

5d $$ \frac{d\left[R\hbox{'}\right]}{dt}=-\left({k}_1\left[M\right]+{k}_5\left[SM\right]\right)\left[R\hbox{'}\right]+{k}_{- 1}\left[MR\hbox{'}\right]+{k}_{- 5}\left[SMR\hbox{'}\right]+\kappa \left[R\right]-\gamma \left[R\hbox{'}\right] $$

Here we also assume the total concentrations of [*R*_*T*_], [*S*_*T*_] and [*M*_*T*_] to be constant.

6a $$ \left[{R}_T\right]=\left[R\right]+\left[R\hbox{'}\right]+\left[MR\hbox{'}\right]+\left[SMR\hbox{'}\right] $$

6b $$ \left[{S}_T\right]=\left[S\right]+\left[SM\right]+\left[SMR\hbox{'}\right] $$

6c $$ \left[{M}_T\right]=\left[M\right]+\left[SM\right]+\left[MR\hbox{'}\right]+\left[SMR\hbox{'}\right]. $$

For the modulator protein to be in detailed balance, the following relations must hold at steady state [41]:

7a $$ \frac{\left[R\hbox{'}\right]\left[M\right]}{\left[MR\mathit{\hbox{'}}\right]}=\frac{\left({k}_{- 1}+{k}_2\right)}{k_1}={K}_M $$

7b $$ \frac{\left[S\right]\left[MR\hbox{'}\right]}{\left[SMR\mathit{\hbox{'}}\right]}=\frac{k_{- 3}}{k_3}={K}_3 $$

7c $$ \frac{\left[R\hbox{'}\right]\left[SM\right]}{\left[SMR\mathit{\hbox{'}}\right]}=\frac{k_{- 5}+{k}_6}{k_5}=K{\hbox{'}}_M $$

7d $$ \frac{\left[S\right]\left[M\right]}{\left[SM\right]}=\frac{k_{- 4}}{k_4}={K}_4 $$

Hence the biochemical rates are constrained by:

7e $$ {K}_3{K}_M={K}_4K{\hbox{'}}_M $$

To characterize the dose-response behavior we consider the concentration of active regulator *R’* as a function of the total amount of signal.

#### Effective class I control parameters

As indicated in Fig. 3a, there are two allosteric modes for an enzymatic modulator: one controls binding, while the other regulates the enzymatic activity. As a result, for an enzymatic modulator, there are two Class I parameters that quantify the cooperativity for each mode. We thus introduce the enzymatic cooperativity defined as:

8a $$ {\alpha}_E=\frac{k_6}{k_2} $$

This parameter is given by the ratio of the rates of enzymatic inactivation of the modulator/regulator complex and the ternary complex of signal, modulator and regulator, *k*_*2*_ and *k*_*6*_ respectively. For enzymatic modulators characterized by *k*_*6*_ *< k*_*2*_, binding of the signal decreases the enzymatic activity of the modulator. This will be referred to as negative enzymatic cooperativity (*α*_*E*_ < 1). On the other hand for *k*_*6*_ *> k*_*2*_ the enzymatic activity of the modulator is elevated by binding the signal, and hence this is termed positive enzymatic cooperativity (*α*_*E*_ > 1). In addition to the enzymatic activity, the concentration of activated (and un-complexed) regulator is also affected by binding interactions, as in the binding modulator system. To describe the effective degree of cooperativity in the binding mode, we introduce the binding cooperativity defined as:

8b $$ {\alpha}_B=\frac{K{\hbox{'}}_M}{K_M} $$

which is given by ratio of the Michaelis-Menten constants of the stimulated modulator *K*_*M*_*’* and unstimulated modulator *K*_*M*_, respectively. For *k*_*2*_ = *k*_*6*_ = 0, i.e. when the modulator does not act enzymatically, the modulator acts as a binding modulator and, as expected, the effective binding cooperativity is equal to the cooperativity *α* of the binding modulator (*α*_*B*_ = *α*).

In a manner analogous to the approach employed for binding modulators, we can define a parameter that relates the relative ligand affinities of the modulator according to:

8c $$ {\beta}_E=\frac{K_3}{K_M}=\frac{K_4}{K{\mathit{\hbox{'}}}_M} $$

#### Effective class II control parameters

In addition, we introduce a set of Class II parameters that otherwise affect the state of the signaling system. Two parameters are defined analogously to their counterparts in the binding modulator model, namely the relative modulator concentration:

9a $$ \mu =\frac{\left[{M}_T\right]}{\left[{R}_T\right]} $$

and the relative regulator dissociation constant, which now reads:

9b $$ {K}_r=\frac{K_M}{\left[{R}_T\right]} $$

In addition, there are two parameters that relate to the enzymatic activation and deactivation of the response regulator in the absence of the signal. The relative enzymatic inactivation rate measures the enzymatic activity of the modulator with respect to the upstream activation and the intrinsic deactivation rate of the response regulator:

9c $$ \iota =\frac{k_2}{\gamma +\kappa}\;. $$

In addition, in the absence of any modulator, the activity of the response regulator is determined by the rates of enzymatic activation and intrinsic deactivation and is given by:

9d $$ {\kappa}_r=\frac{\kappa }{\gamma +\kappa}\;. $$

This parameter also determines the regulator activity in absence of the modulator ${\left[R\hbox{'}\right]}_{M_T=0}={\kappa}_R\left[{R}_T\right]$. Note that all Class II parameters in our model are now dimensionless.

### Features used to characterize the response behavior

#### Dynamic output range

Perhaps the most important characteristic of the steady-state response is the response amplitude. For monotonically increasing or decreasing dose-response curves, the (relative) response amplitude is identical to the (relative) dynamic range of the output of the signaling system, which is expected to be strongly correlated with the ability of the system to transmit information about the environment [42]. We define the response amplitude of a modulator signaling system as the difference between the basal concentration of the free (active) response regulator when no signal is present and the level attained when the signal is present at saturating concentrations. For convenience, we normalize the response amplitude to the total amount of regulator, i.e.:

10 $$ A=\frac{\left[R\right]\left(\left[{S}_T\right]\to \infty \right)-\left[R\right]\left(\left[{S}_T\right]=0\right)}{\left[{R}_T\right]} $$

#### Qualitative action of the signal

According to our definition, *A* is bounded by −1 and +1, and hence an explicit expression for *A* can also be used to determine the qualitative action of the signal. For *A* > 0 the signal counteracts the action of the modulator (i.e. the signal acts as an inverse agonist) and the pathway is activated by the signal. Conversely, for *A* < 0 the signal enhances the action of the modulator (i.e. the signal acts as an agonist).

#### Robustness to signaling context

We consider a modulator to function robustly if the qualitative response behavior does not change as a function of Class II parameters. To computationally distinguish between robust and non-robust systems, we introduce the “response entropy” *H* defined as:

11 $$ H(p)=-p{ \log}_2(p)-\left(1-p\right){ \log}_2\left(1-p\right) $$

Here *p* is the frequency of Class II parameter sets for which addition of the signal results in an increase in the amount of active response regulator. Correspondingly, (1-*p*) is the frequency of deactivating responses. *H* is maximal when half of the parameter sets result in activation and half of them result in deactivation of the response regulator. For *H* = 0, the stimulation of the qualitative response (i.e. activation or inhibition) is robust and independent of Class II parameters, while a positive response entropy (*H* > 0) indicates a context-dependent response.

#### Operating regime (EC_50_)

We use the *EC*_*50*_, i.e. the effective input signal which is required to achieve a half-maximal response of the regulator, to characterize the operating regime of a modulator signaling system. For graded response curves the *EC*_*50*_ is a useful way to quantify the input regime to which the signaling system responds most effectively. For switch-like response curves the *EC*_*50*_ denotes the threshold concentration required to trigger the switch.

#### Graded and switch-like response (Hill coefficient)

To distinguish between graded and more switch-like response curves we use the Hill coefficient *h*. To this end we compute the elasticity coefficient defined as:

12 $$ \varepsilon \left({S}_T=E{C}_{50}\right)={\left.\frac{d \log {R}_n}{d \log {S}_T}\right|}_{S_T\to E{C}_{50}} $$

Where is the normalized dose-response ${R}_n=\frac{\left[R\right]\left(\left[{S}_T\right]\right)-\left[R\right]\left(\left[{S}_T\right]=0\right)}{\left[R\right]\left(\left[{S}_T\right]\to \infty \right)-\left[R\right]\left(\left[{S}_T\right]=0\right)}$ curve. The Hill coefficient *h* is then given by *h* = 2* *ε* (*S*_*T*_ = *EC*_*50*_) [42].

Response curves with *h ≤ 1* are considered to be graded, while *h > 1* denote ultrasensitive, more switch-like behaviors.

## Results

### Signaling via binding modulators

#### Switchable binding modulators can implement various response behaviors

**Fig. 4:**
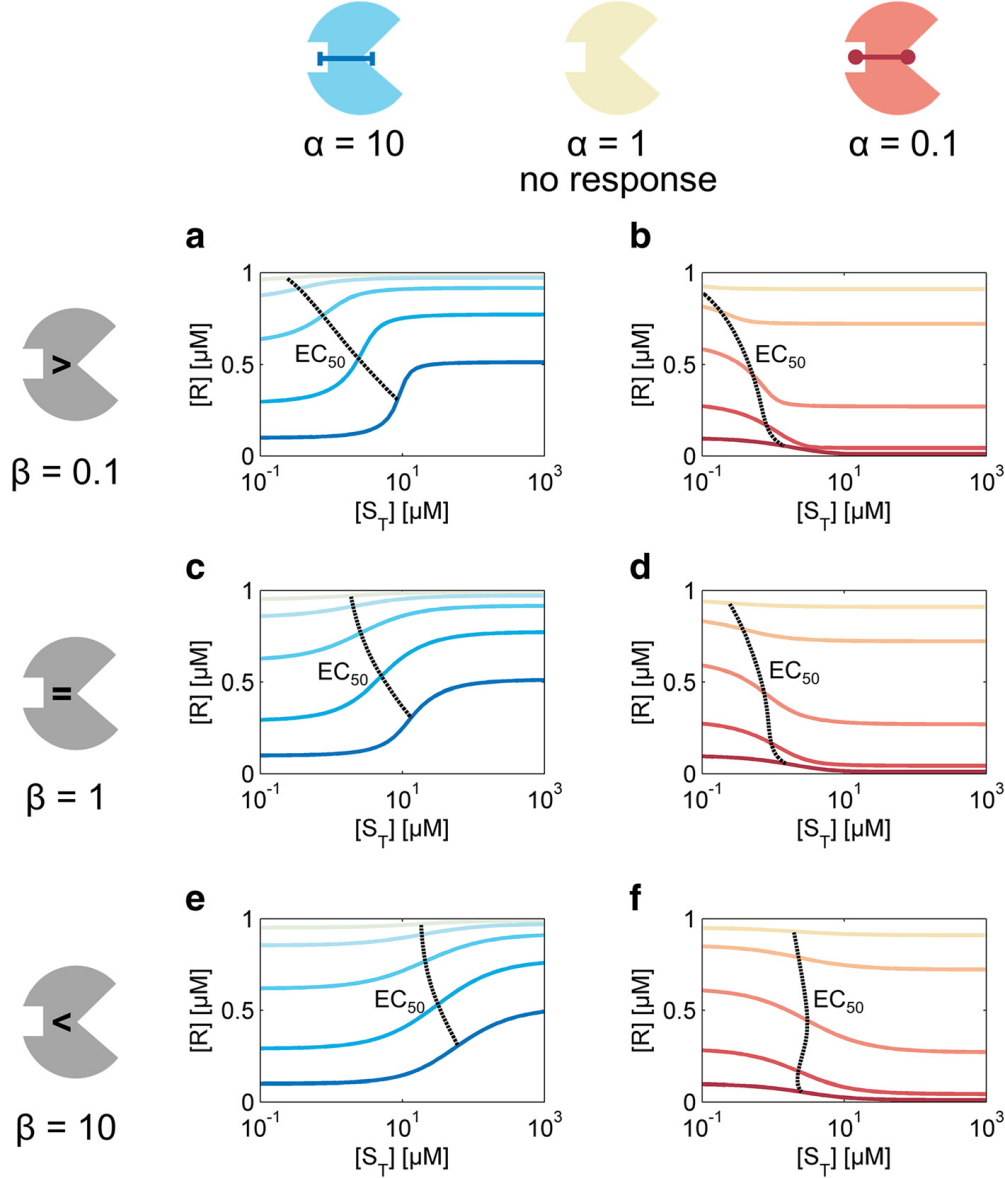
Switchable binding modulators can implement various response behaviors. The physiochemical properties of a modulator protein are controlled by the cooperativity *α* and the relative affinity of the two allosteric sites for their respective ligands *β*. In the cartoons, the regulator binding site is indicated by the open triangle, the signal binding site by the open square. Panels A–F show dose-response curves for the six different allosteric binding modulator proteins schematically depicted in the respective cartoons. Note, that *α = 1* denotes a modulator molecule without cooperativity and thus it does not function as a signaling receptor (no response). For each receptor, a set of response curves is shown that results from varying total modulator concentration [M_T_] at fixed receptor concentration [R_T_]. Color intensity in each panel increases with stepwise increase in μ = [M_T_]/[R_T_] from 0.1, 0.32, 1, 3.2, 10. For all curves we set K_1_ = [R_T_] =1 μM. Blue lines denote inverse agonistic signaling schemes and the red lines agonistic schemes. The dotted lines indicate the shift in the EC_50_ as a function of *μ*. **a** *α* = 10, *β* = 0.1, **b** *α* = 0.1, *β* = 0.1, **c** *α* = 10, *β* = 1, **d** *α *= 0.1, *β* = 1 **e** *α* = 10, *β* = 10, **f** *α* = 0.1, *β* = 10

To obtain insight into the response characteristics of a binding modulator system we first simulated the reaction network motif depicted in Fig. 2b for different parameters sets to calculate the respective dose-response curves. To this end we considered six modulator proteins, which differ in their cooperativity *α* and the relative affinities of the two effector sites for their respective ligands given by *β* (Fig. 4). Since we wished to extensively explore the behavior of the model, we made no a priori assumptions with regard to the parameter values. Thus, our analysis includes both Rap-like modulator proteins, which exhibit negative cooperativity (*α* > 1, right column), but also putative modulator proteins that engage in positively cooperative interactions (*α* < 1, left column). In the latter case, the signal enhances interaction with the regulator. We considered a modulator protein that preferentially binds to the signal (*β* > 1, top), one that favors binding to the regulator (*β* < 1, bottom) and one with equal affinity to both interaction partners (center). Moreover, for each modulator protein, we studied the effect of the signaling context by varying the concentration of the modulator over two orders of magnitude. This results in a series of response curves in each case.

As shown in Fig. 4, we find that the overall spectrum of responses includes agonistic and inversely agonistic behaviors, with both graded and more switch-like curves. Furthermore, there is considerable variability in the response amplitude and in *EC*_*50*_ level. Hence SAMPs can be used to implement a diverse set of response behaviors by varying the physicochemical properties of the modulator (Class I parameters) and the signaling context (Class II parameters), respectively. We next investigated the parametric dependencies of key features of the input–output relationship, using (a) the sign and the magnitude of the response amplitude to characterize qualitative response and output dynamic range, (b) the Hill coefficient to characterize the shape of the does-response curve and distinguish between graded and more switch-like responses, and (c) the *EC*_*50*_ to characterize the input operating regime of the signaling system.

#### Context-dependent and -independent features of the signaling response

**Fig. 5:**
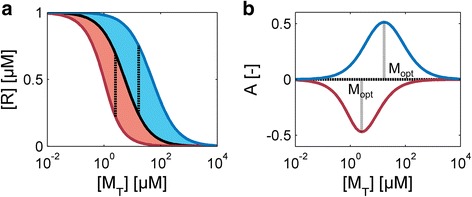
The modulator concentration that is optimal for signaling. **a** Titration curves of the free regulator [R] as a function of the total modulator concentration [M_T_] in the absence of the signal (black line) and at saturating signal concentrations. For positive cooperativity the titration curve shifts to the left (*α* = 0.1, red line) and for negative cooperativity to the right (blue line, *α* = 10). The shaded red (blue) regime indicates all possible signaling outputs that can be obtained by varying S in each case. The difference between the two titration curves (vertical line) determines the output dynamic range at a fixed modulator concentration. Other parameters: [R_T_] = 1 μM for K_1_ = 5 μM. **b** The relative response amplitude A as a function of the modulator concentration for the two systems shown in **a**. The amplitude reaches an optimum A_opt_ at a particular M_opt_ in each case (vertical line)

The dose-response curves for binding SAMPs are monotonically increasing or decreasing functions of the signal, so that the response amplitude *A* is given by the difference between the basal level of *R* in the absence of *S* and the plateau-level that is reached at saturating signal concentrations. The basal level varies as the response regulator is titrated by the modulator as a function of the ratio *μ* of their respective concentrations (Fig. 5a). Analogously, at saturating signal concentrations, the titration curve is shifted to lower (higher) modulator concentrations for proteins displaying positive (negative) cooperativity. The difference between the two titration curves in each case determines the relative output dynamic range *A*, which is given by:

13 $$ A=\frac{1}{2}\left({K}_{R1}\left(1-\alpha \right)-\sqrt{{\left(\mu +1+{K}_{R1}\right)}^2-4\mu }+\sqrt{{\left(\mu +1+\alpha {K}_{R1}\right)}^2-4\mu}\right) $$

A derivation of (Eq. 13) is given in Additional file 1. Hence, *A* depends on three parameters: the cooperativity *α*, the relative modulator dissociation constant *K*_*R1*_ and the modulator-to-regulator ratio *μ*.

##### The qualitative response behavior is robust to changes in the signaling context

As seen from Eq.13, whether the signal acts as an agonist or inverse agonist is controlled solely by the cooperativity α. This was previously demonstrated for the ternary complex model [37] and, as a result of shared structural elements of the two models, it also applies to the binding modulator model. If the cooperativity is negative (*α* > 1), binding of the signal inhibits the binding of the regulator and vice versa. Regulator and signal then compete for modulator binding, which resembles the case in which the signal binds at the regulator binding site, i.e. when the signal is an orthosteric ligand. Thus in this case, the signal acts as an allosteric inverse agonist since the regulator/modulator complex is dissociated and the concentration of free regulator increases. On the other hand, for positive cooperativity (*α* < 1), binding of the signal enhances the affinity of the modulator for the regulator and vice versa. Hence, the addition of the signal leads to formation of the ternary complex *SMR*. This in turn *decreases* the concentration [*R*] of the free regulator. Therefore, for systems with positive cooperativity, the signal acts as a repressor, i.e. the signal is an allosteric agonist.

##### Existence of a modulator concentration optimal for response amplitude

Notably, for a given modulator protein, the response amplitude (Eq.13), as plotted in Fig. 5b, displays a non-monotonic dependence on the modulator-to-regulator ratio *μ*. If the modulator concentration is too low the regulator will be free, irrespective of the presence of the signal. On the other hand, if the modulator concentration is too high, the regulator will always be associated with the modulator. In the intermediate concentration regime the signal can free the regulator from the regulator-modulator complex when bound to a modulator with negative cooperativity or, conversely, can trap the regulator in the SMR complex by interacting with a modulator with positive cooperativity. In order to maximize the output dynamic range the (total) modulator and the regulator concentration must fulfil the following relationship:

14 $$ \left[{M}_{opt}\right]=\left[{R}_T\right]+\sqrt{K_1{K}_5} $$

Hence, the modulator must generally be present in excess over the regulator to meet the optimum. Furthermore, the weaker the interactions of the modulator, regulator and signal (i.e. the larger the product of the dissociation constants *K*_1_* K_*5*_), the more modulator is required. Thus the signaling context, as quantified by *μ*, is a key parameter that will affect the ability of the signaling system to transfer information. Moreover, the Hill coefficient *h* can also be altered by varying *μ*. For Rap-like modulator proteins with negative cooperativity, the Hill coefficient increases as a function of *μ* and, at the same time, the *EC*_*50*_ shifts to higher input levels. Indeed, *μ* can even shift the response from a graded (*h ≤ 1*) to an ultrasensitive induction (*h > 1*). For modulators with positive *α*, the model predicts even more complex dependencies on *μ* (see Fig. 4). To summarize, while variations in signaling context can lead to substantial variability in the output behavior of binding modulator systems, they cannot change their qualitative response behavior.

#### Control of the response behavior by the physicochemical properties of a binding modulator

To systematically analyze how the physicochemical properties of a SAMP can affect signal transduction, we assumed that the concentration of each modulator is set at the level that optimizes the response amplitude (i.e. *μ* → *μ*_opt_). Hence the system always operates under conditions which are favorable for information transfer, which allows us to focus on the effects of Class I parameters.

##### Signaling amplitude

**Fig. 6:**
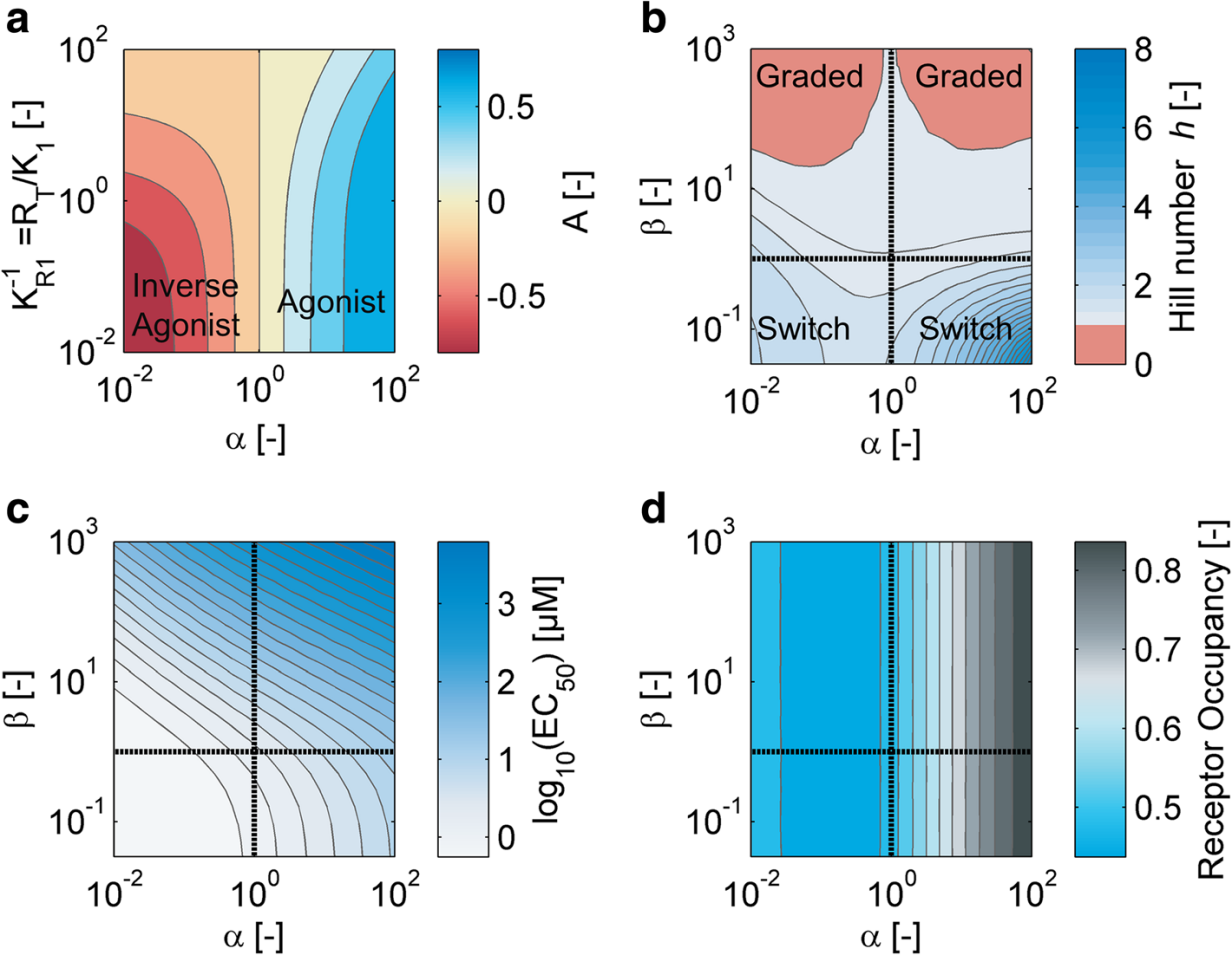
Variations in the molecular properties of modulator proteins alter key features of response behavior. The concentration of each modulator protein is set to the level that optimizes the output dynamic range. **a** Output dynamic range: A_opt_ as a function of the cooperativity α and the inverse of the relative regulator dissociation constant K_R1_. **b** Graded and switch-like responses: The Hill coefficient computed from the normalized response curves differentiates between modulator proteins which produce graded and switch-like responses. **c** EC_50_ and **d** fractional receptor occupancy at the EC_50_, plotted as a function of the cooperativity *α* and the ratio of the ligand affinities, *β*. In panels **b** and **c** we assumed K_1_ = [R_T_] = 1 μM

Under the assumption that the system operates with optimal *μ*, the response amplitude is given by

15 $$ {A}_{opt}=\frac{1}{2}\left({K}_{R1}\left(1-\alpha \right)-\left(1-\sqrt{\alpha}\right)\sqrt{K_{R1}\left({K}_{R1}{\left(1+\sqrt{\alpha}\right)}^2+4\right)}\right) $$

and is plotted in Fig. 6a. A strong cooperative effect generally favors a wide dynamic output range. Moreover, the affinity of the modulator for the regulator should be rather weak (large *K*_*R1*_), i.e. a high dissociation constant *K*_*1*_ or a small total regulator concentrations [*R*_*T*_] will tend to broaden output dynamic range.

##### Graded and ultrasensitive responses

In general, there are several ways in which a cellular response can be induced as a function of the signaling input. For example, for certain two-component systems the output can be modulated in a graded fashion while others display switch-like properties, either with or without hysteresis, depending on their exact architecture [43–46]. We thus computed the Hill coefficient *h* as a function of *α* and *β* to gain insight into how the properties of the modulator protein shape the response function (Fig. 6b). We find that modulator proteins that display a high affinity for their cognate signal (i.e. *β* is low) exhibit ultrasensitive response curves, while SAMPs that display strong cooperativity but preferentially bind to the response regulator (large *β*) tend to show graded responses. Note that, since our model does not support bistable outputs, switching always occurs without memory.

##### EC_50_ and fractional receptor occupancy

The molecular properties of the modulator protein also affect the sensitive input regime_._ For Rap-like binding modulators with a more switch-like response characteristic, strong cooperative interactions not only increase the response amplitude and the Hill coefficient of the response curve, but also shift the threshold concentration required to trigger the switch to higher signal levels. This is evidenced by rising *EC*_*50*_ levels with increasing *α*. On the other hand, for modulators that implement graded responses, the *EC*_*50*_ can be tuned by varying *β* as well as *α* (Fig. 6c). Finally, fractional receptor occupancy, i.e. the fraction of the modulator protein bound to the signal at the *EC*_*50*_, increases with increasing *α* for Rap-like modulators, but shows a biphasic behavior for modulators with positive cooperativity (Fig. 6d).

### Signaling via enzymatic modulators

#### Context-dependent and -independent features of the signaling response

**Fig. 7:**
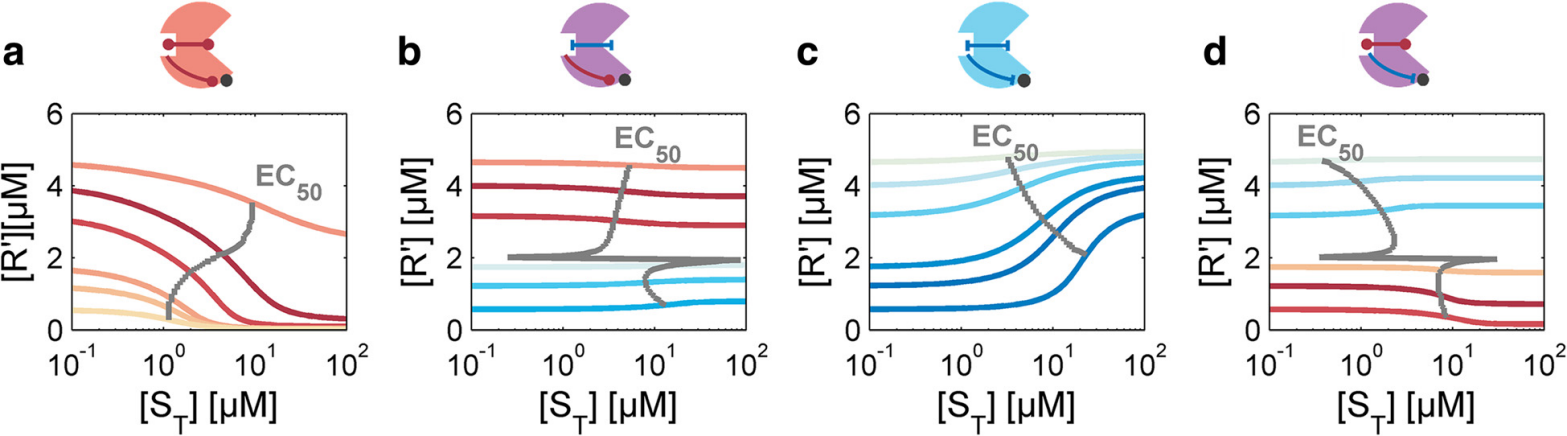
Dose-response curves for enzymatic SAMPs with different allosteric properties. For coherent proteins depicted in red (blue) enzymatic and binding cooperativity are both positive (negative). The purple proteins are incoherent modulators, in which the two allosteric effects oppose each other. **a** *α*_*E*_ = 10 *α*_*B*_ = 0.1 **b** *α*_*E*_ = 10 *α*_*B*_ = 10 **c** *α*_*E*_ = 0.1 *α*_*B*_ = 10 **d** *α*_*E*_ = 0.1 *α*_*B*_ = 0.1. The different curves result from variations in the total modulator concentration [M_T_] = (0.5, 1.5, 3, 7, 10, 20) μM in each case. Red (blue) lines denote antagonistic (inverse antagonistic) responses. The color intensity of each line correlates with the magnitude of the output dynamic range in each case. Other parameters: [R_T_] =10 μM, *κ = γ* = k_−1_ = k_2_ = k_−3_ = k_−4_ = k_−5_ = 1 s^−1^ and k_1_ = k_3_ = 1 s^−1^ μM^−1^. k_4_, k_5_, k_6_ are determined by the allosteric parameters and the requirement for detailed balance

We next conducted a similar analysis of enzymatic modulator systems as depicted in Fig. 3, in order to identify shared principles and differences between the two signaling modes. One important distinction between enzymatic and binding modulator proteins is that enzymatic modulators have two allosteric modes, one for binding as quantified by *α*_*B*_ and one affecting the enzymatic reaction as quantified by *α*_*E*_. Thus, there exist four conceivable operational models for the design of enzymatic SAMPs. The allosteric effects on binding and enzymatic activity could act in the same direction, i.e. both activate or repress, respectively, or alternatively they could oppose each other. We refer to the former as *coherent* and the latter as *incoherent* SAMPs. We thus simulated the dose-response curves for each type by varying the concentration of the modulator in each case (Fig. 7). In accordance with intuitive expectation, for coherent modulators we found that the signal always acts as an inverse agonist for enzymes with negative cooperativity and as an agonist for modulators exhibiting positive cooperativity. In contrast, for incoherent modulators the response is weaker (i.e. results in lower response amplitudes). Moreover, the responses change from repression to activation and vice versa depending on the amount of modulator protein present. Thus, at least for certain enzymatic modulators, the qualitative response behavior is dependent on the signaling context. This distinguishes enzymatic from binding modulators.

##### Output dynamic range

To investigate the response behavior further, we derived an algebraic expression for the response amplitude. By using the effective Class I and Class II parameters defined above (see Models), we were able to derive the fairly compact mathematical expression:

16 $$ A=\frac{1}{2}\left({K}_r\left(1-{\alpha}_B\right)+\mu \iota \left(1-{\alpha}_E\right)-\sqrt{4{K}_r{\kappa}_r+{\left({K}_r+\mu \iota +{\kappa}_r\left(\mu -1\right)\right)}^2}+\sqrt{4{\alpha}_B{K}_r{\kappa}_r+{\left({\alpha}_B{K}_r+{\alpha}_E\mu \iota +{\kappa}_r\left(\mu -1\right)\right)}^2}\right) $$

A derivation of (Eq. 14) is given in Additional file 1. We note that, as long as the parameters fulfil the relationships defined by Eqs. (7), the dose-response curves are monotonic functions of the signal. Hence, *A* again reflects the output dynamic range, which is therefore a complicated function of various Class I and Class II parameters.

##### For certain classes of SAMPs signals act both as agonists and inverse agonists

**Fig. 8:**
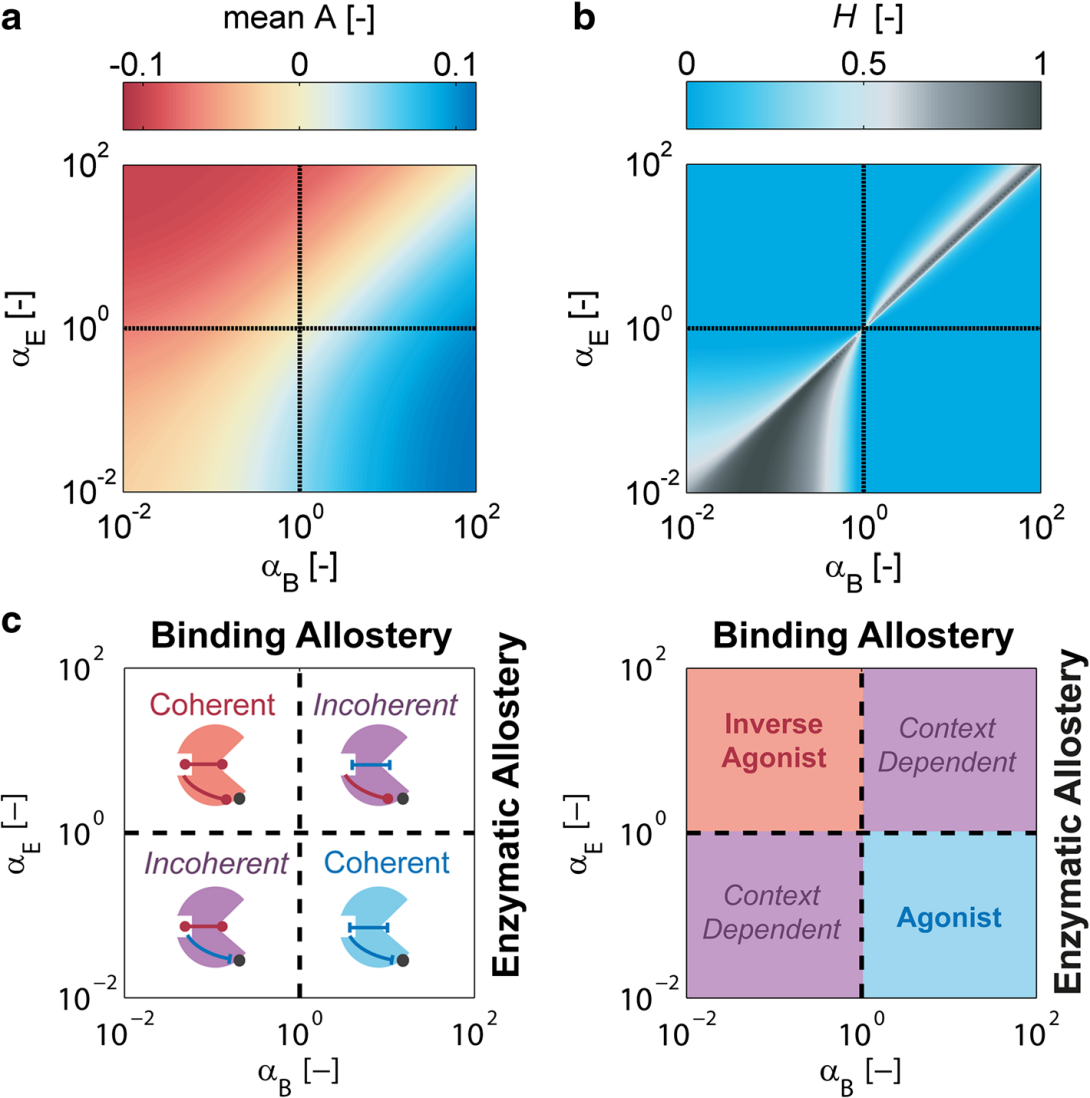
The allosteric class of an enzymatic SAMP affects its response behavior. **a** Mean response amplitude is plotted as a function of cooperativity by averaging over 10^4^ different sets of Class II parameters. *K*_1R_, *ι* and *μ* were varied between 0.1 and 10, and *κ*_*R*_ in the range of 0.01 to 1 by Latin hypercube sampling. **b** Corresponding response entropy *H.* **c** Cartoon summarizing the qualitative response behavior of the different classes of enzymatic modulators

To investigate whether the findings derived from the simulations in Fig. 7 apply more generally, we computed the mean response amplitude < *A* > as a function of the cooperativity parameters ${\alpha}_E$ and ${\alpha}_B$ by averaging over 10^4^ different parameter sets for the Class II parameters (Fig. 8a). Figure 8b shows the corresponding qualitative response entropy *H*, defined in Eq.(7) as a measure for the sensitivity of the qualitative response to changes in the signaling context. The results clearly demonstrate that coherent SAMPs show a robust qualitative response (*H = 0*), signals act as agonist or inverse agonist, respectively and the response amplitude tends to increase as the strength of the cooperative effects increases. In contrast, incoherent SAMPs may switch their qualitative behavior depending on the signaling context (*H > 0*), and show smaller average signal amplitudes (Fig. 8a). Hence, incoherent SAMPs are less capable of facilitating information transfer, and, most probably for this reason, are selected against in nature, and are not considered further here.

##### Modulator concentration optimal for response amplitude correlates with the kinase activity

**Fig. 9:**
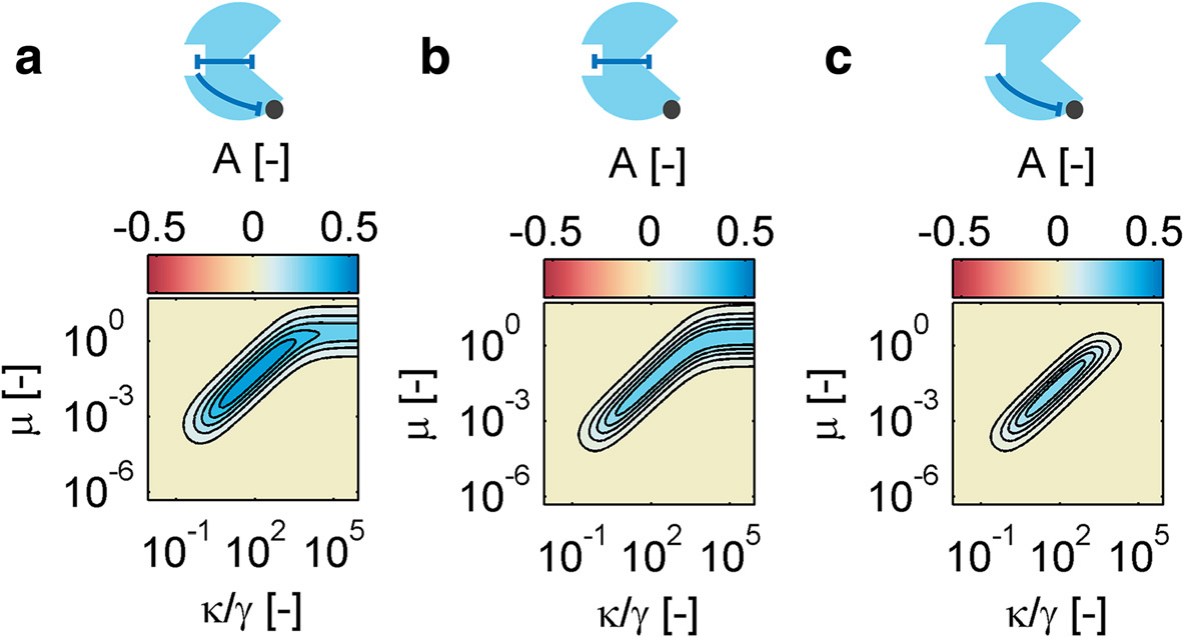
Conditions optimal for signaling amplitude. Relative response amplitudes A for three Rap-like SAMPs are plotted as a function of the modulator-to-regulator ratio *μ* and the relative kinase activity *κ/γ*. **a** Coherent enzyme with *α*_*E*_ = 0.37 and *α*_*B*_ = 5. **b** Enzyme without enzymatic allostery (*α*_*E*_ = 1). **c** Enzyme without binding allostery (*α*_*B*_ = 1). Other parameters: [R_T_] = 2 μM, K_M_ = 1.2 μM, k_2_ = 0.72 s^−1^, *γ* = 1.2*10^−4^ s^−1^

We next investigated how the signaling context affects the output dynamic range. To this end we considered three Rap-like enzymatic inhibitors: a coherent enzymatic modulator, an enzymatic modulator which acts by binding allostery only, and an enzymatic modulator which acts by enzymatic allostery alone. Figure 9 shows the corresponding output dynamic range as a function of *μ* and the relative kinase activity *κ/γ*. In analogy to the case of binding modulators, for enzymatic modulators there likewise exists a concentration for the modulator protein that is optimal for response amplitude. Its value depends on the concentration of the regulator via *μ* and on the relative kinase activity *κ/γ*. With increasing kinase activity, the modulator concentration must rise roughly in proportion in order to achieve the response optimum in all three cases. When the kinase activity saturates the pathway, the optimal modulator concentration saturates at a level that roughly corresponds to the amount that would be required by a non-enzymatic binding modulator (Fig. 9a and b). Lesser amounts of an enzymatic modulator than of a binding modulator are required to achieve the response optimum. This is best illustrated by an enzymatic modulator in which the allosteric effect only acts on binding. Here, we find that the optimum is given by

17 $$ {\mu}_{opt}=\frac{1}{1+\frac{k_2}{\kappa }}\left({\mu}_{opt,B}+\frac{\gamma }{\kappa}\left({\mu}_{opt,B}-1\right)\right) $$

where *μ*_*opt,B*_ is the optimal value for a corresponding binding modulator given in Eq.14 with *α* being replaced by *α*_*B*_ and *K*_*R1*_ by *K*_*r*_. With increasing kinase activity *μ*_*opt*_ increases and saturates at the level of *μ*_*opt,B*_. For enzymatic modulators in which the allosteric effect acts only on the enzymatic activity, our model predicts the existence of a global optimum (Fig. 9c).

#### Control of response behavior by the molecular properties of an enzymatic modulator

**Fig. 10:**
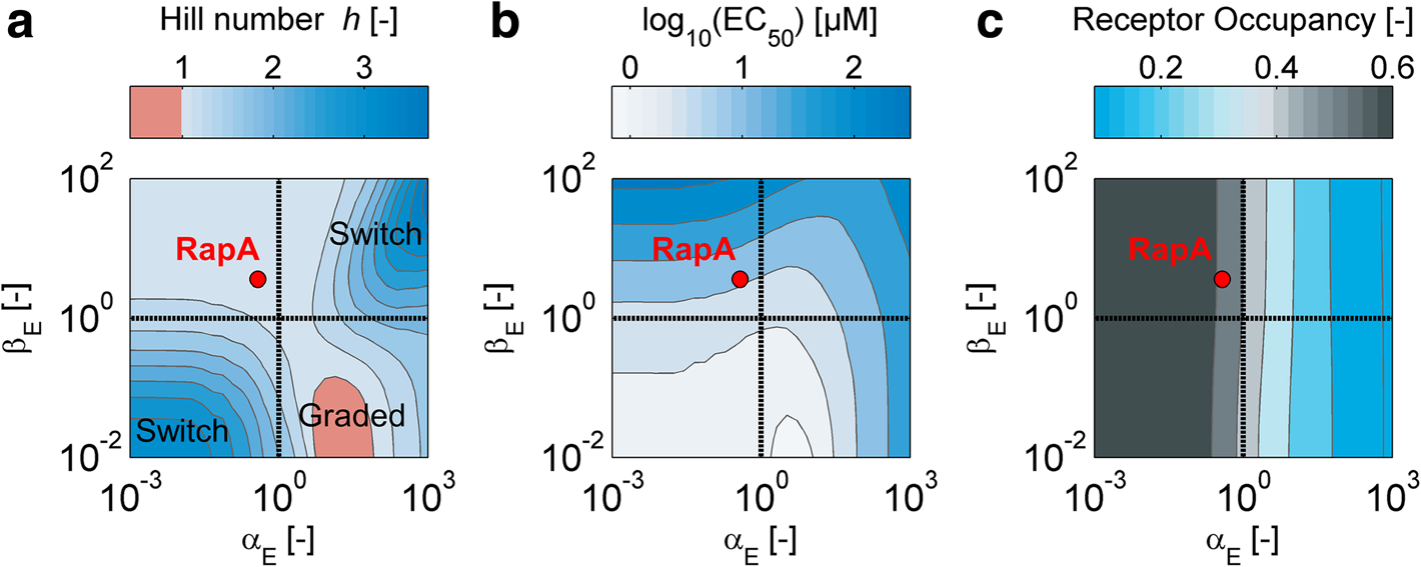
The molecular properties of an enzymatic receptor shape the cellular response. The concentration of each enzymatic modulator protein is set to the level that optimizes the output dynamic range. **a** Graded and switch-like responses: The Hill coefficient computed from the normalized response curves differentiates between modulator proteins which produce graded and switch-like responses. **b** EC_50_ and **c** fractional receptor occupancy at the EC_50_ as a function of the enzymatic allostery α_E_ and the relative affinity ${\beta}_E$. Other parameters: [R_T_] = 10 μM, *κ* = k_−1_ = k_2_ = k_−3_ = k_−4_ = k_−5_ = 1 s^−1^, *γ* = 0.1 s^−1^ and k_1_ = k_3_ = 1 s^−1^ μM^−1^. k_−3_, k_4_, k_5_, k_6_ are given by the allosteric parameters and the requirement for detailed balance. The red dot indicates the location of the RapA-PhrA system from *B. subtilis* in the parametric design space of SAMPs

To systematically analyze how the molecular properties of an enzymatic SAMP affect signaling, we again assumed that the protein operates under conditions that are optimal for signaling amplitude, i.e. we optimized [*M*_*T*_] and *κ* for each protein under consideration. We focused on enzymatic modulators with *α*_*B*_ = 1. We explored the design space of proteins by sampling *α*_*E*_ and *β*_*E*_, and studied the effect on the Hill number *h*, the *EC*_*50*_ and receptor occupancy. Like binding modulators, enzymatic modulators could in principle implement both switch-like and more graded responses (Fig. 10a). For Rap-like modulators with *α*_*E*_ < 1 a high signal affinity (*β*_*E*_ < 1) results in more switch-like behavior. Moreover, the *EC*_*50*_ shifts with increasing *β* to higher levels (Fig. 10b). Interestingly, for modulators in which ligand binding activates enzymatic activity, our model predicts that switch-like response requires a preference for regulator binding. Here the *EC*_*50*_ is relatively insensitive to changes in *β*_*E*_, but responds to changes in *α*_*E*_. Finally, the receptor occupancy at *EC*_*50*_ increases with decreasing *α*_*E*_ (Fig. 10c).

## Discussion

Raps from the genus *Bacillus* have been studied from genetic and biochemical perspectives for decades [47]. However, only very recently, thanks to the elucidation of the structures of several key molecular complexes, has it become apparent that they function as monomeric allosteric receptor proteins. This opens a new dimension for research. In particular, the time has come to begin integrating biochemical and structural data and physiological insights with the help of mathematical models to advance our understanding of SAMP-based signaling and exploit it for practical applications. Here we have used two mathematical models to describe signal transduction via SAMPs, both of which could facilitate such integrative studies. For our models we adopted an allosteric viewpoint, and characterized the receptor with the help of effective (allosteric) parameters in order to analyze quantitatively how the molecular properties of SAMPs affect signal transduction.

Except for the case of RapA from *B. subtilis* [48–50] quantitative biochemical data on SAMPs are still limited. For RapA our (allosteric) parameter estimates read as follows: *α*_*B*_ = 1, *α*_*E*_ = 0.37 and ${\beta}_E$ = 3.6. The position of the RapA in the design space of receptors is indicated in Fig. 10, from which we can read off the predicted response behavior, provided the signaling network operates under conditions that are optimal for information transfer (i.e. when the upstream kinase is moderately active and modulator concentration is appropriately matched to the regulator concentration). The model predicts that the concentration required for 50 % receptor occupancy is approximately equivalent to the predicted *EC*_*50*_ and that RapA should give rise to a fairly graded response function upon stimulation with PhrA. The latter inference is consistent with its ability to progressively control sporulation frequency—the final physiological output that is controlled by the RapA-PhrA signaling system—over a wide range of concentrations [51].

Interestingly, for RapA, our model predicts little—if any—allosteric inhibition of ligand binding. Given that many Raps function as binding modulators, and thus must have strong allosteric interactions for ligand binding, this finding might be surprising. Also, our model does not offer any obvious explanation why nature should select against cooperative effects on ligand binding in enzymatic SAMPs. However, recent structural studies of Rap proteins suggest at least a partial answer to this puzzle. Raps employ two distinct interfaces to interact with response regulators. Binding modulators interact with the C-terminal output domain of the response regulator, while enzymatic modulators interact with the N-terminal input domain [21–24]. Thus the binding and the enzymatic signaling mode might have evolved independently—but perhaps simultaneously—in the ancestral SAMP. It is intriguing to speculate that this protein might once have been a coherent enzymatic SAMP that interacted with one response regulator using both interaction interfaces (and not with two distinct regulators, like the “modern” bifunctional SAMPs [16]).

During the course of evolution, Rap-Phr systems have spread across and within genomes [52]. Frequently, multiple homologs are present in a cell and regulate important, but diverse, phenotypes. Different phenotypes may have differential regulatory needs to enable the organism to adapt properly. For example, some cellular responses turn on in a graded fashion, while others show switch-like behaviors. From a mechanistic point of view, comparable regulatory outcomes could theoretically be generated in diverse ways. Our study suggests that, in principle, SAMPs could enable bacteria to implement a diverse spectrum of response behaviors by evolutionary adjustment of their physicochemical properties. In particular, our model predicts that the relative affinity of the two effector binding sites plays a key role in shaping and tuning the response function. In this context, it would be very interesting to investigate experimentally the extent to which Rap proteins have diverged in this way, and how such changes in their molecular properties correlate with the underlying phenotypic response behavior.

In addition to the SAMP’s molecular properties, our study suggests that the signaling context is very important for shaping the response behavior. That the outcome of signaling is dependent not only on the molecular properties of the receptor but also on other cellular factors is a very common observation [53]. On the other hand, for certain two-component systems a balanced biochemical set-up allows them to generate robust input-output relationships with respect to fluctuations in the concentrations [54]. In addition, compensatory mechanisms could alleviate cell-to-cell variability and lead to more robust cellular behavior [55–57]. However, since the physiological processes controlled by SAMPs often display a heterogeneous output across the population [31, 58], we speculate that the context dependence of the signaling responses mediated by SAMPs might actually be utilized to diversify the population.

With respect to information processing, our model predicts that here again the signaling context plays a dominant role. In particular, there exists a concentration of SAMPs that is optimal for signal amplitude and is thus expected to be optimal for transferring information from the receptor to the response regulator. For binding SAMPs this optimal concentration is always higher than the concentration of response regulator. In contrast, much lower protein levels are typically required for optimal signaling mediated by enzymatic modulators, especially if the activation by the upstream kinase is still low. This design rule might explain the start codon preferences for different Raps. While Raps that function as binding modulators (e.g. RapC, F, D, G, K in *B. subtilis*) use the canonical start codon, translation of many Rap phosphatases (e.g. RapA, E, H, I in *B. subtilis*) is attenuated by the use of a non-optimal start codon. In addition, in order to retain sensitivity to the signal, enzymatic SAMPs would have to adjust their levels as the kinase activity changes. This would also shift the *EC*_*50*_ levels. This might at least partially explain why the transcription of Rap phosphatase genes (and their cognate signaling peptides) is so tightly regulated [59, 60] and shows such complex dynamics [31].

Our models were built with a focus on facilitating a better understanding Rap-signaling in *Bacilli.* However, in bacteria many molecules exist which interact with response regulators. The most well-known modulators are the CheY-like phosphatases that control the chemotaxis pathway in many Gram-negative and Gram-positive bacteria [61] and the Spo0E-like phosphatases that act onto the sporulation phosphorelay in *Bacillus subtilis* [62]. It has often been speculated that the enzymatic activity of these phosphatases is allosterically controlled by cellular signals (and thus they may acts as SAMPs) although definite experimental proof is to the best of our knowledge still lacking [62]. On the other hand, the CadC-one-component system in *Escherichia coli* might be controlled by SAMP-signaling using a binding modulator mode. The Cad-system contributes to pH-homeostasis via a pH-sensitive ToxR-like receptor CadC that directly activates transcription. CadC is co-regulated by lysine via the lysine permease LysP which binds to CadC and thereby inhibits transcription in a lysine-dependent and presumably allosteric manner [63]. Thus, applying the terminology of our model, LysP might act as a lysine-switchable binding modulator. Interestingly, the ability of the Cad-system for properly integrating the lysine signal depends on maintaining a specific ratio between LysP and CadC that is biased towards LysP (“balance model”) [64, 65]. This observation is consistent with the prediction of our model that optimal signal transduction via SAMPs requires the modulator and the response regulator to be present at a particular ratio μ = M_T_/R_T._ Although the oligomeric state of the LysP-CadC complex is not yet known, in case LysP and CadC form a heterodimer, Eq. (14) should apply and could be used to infer on the dissociation constants of the different LysP-CadC complexes.

Per definition SAMPs “modulate” signal transduction and thus the signals that are transduced via SAMPs will be “integrated” with other signals that control the activity of the response regulator. Understanding how the different signals are integrated on the level of the response regulator [31] and decoded on the level of the promoter [66] is an important subject for future experimental and theoretical study. Recent theoretical work on two-component systems and phosphorelays has shown that these systems alone are capable of generating diverse response behaviors [33, 43–46, 67, 68], which are not captured by the simplified treatment of the phosphorylation/de-phosphorylation cycle in our model. To fully understand signaling via SAMPs—especially enzymatic SAMPs—we expect that our models will have to be integrated with more detailed models of phosphorylation-based signal transduction systems.

## Conclusions

To conclude, we have introduced the first kinetic models based on ordinary differential equations designed specifically to study signal transduction by SAMPs. We showed quantitatively how the molecular properties of the receptor (i.e. its allosteric and biochemical functions) and other relevant factors (e.g. relative concentrations of signaling components) control the response behavior of the signaling system. We find that appropriate tuning of the molecular properties of the receptor would enable SAMPs to implement versatile dose-response behaviors. On the other hand, the response is also shaped substantially by cellular factors, suggesting that SAMPs may generate rather variable outputs in a population of “noisy” bacteria. Moreover, for each SAMP, we identified conditions that optimize information transfer from the receptor to the response regulator. We expect that our model will stimulate the development of biomedical and biotechnological applications in the future. Interference with bacterial signaling—by targeting Raps—could lead to alternative antimicrobial treatments. It is worth mentioning here that Rap genes have recently been discovered on a plasmid in a multi-resistant Gram-negative bacterium [69]. Our model provides a useful foundation for future pharmacological studies of SAMPs. In addition, SAMPs also hold promise for use as scaffolds for the design of ligand-switchable affinity reagents [23] and the engineering of novel interactions into bacterial signaling systems [21–24]. The design rules revealed by our study could thus facilitate the rational design of synthetic SAMPs and cellular signaling systems in the near future.

## Methods

### Simulations of dose-response curves

All numerical calculations were performed with Matlab 2011b (MathWorks Inc.). To derive the steady state dose-response curve the models were numerically integrated to steady state at a fixed level of the total amount of signal [S_T_] with the otherwise indicated parameters using the ode15s solver. To verify the steady-state condition the fsolve function was used. The response amplitude was compared to the explicit mathematical expressions given by Eq. (13) and Eq. (16), respectively and found to agree. The mean response amplitude was determined by averaging over 10^4^ different amplitude values computed from Eq. (16) for randomly chosen Class II parameter sets, which were chosen from Latin hypercube sampling. Robustness was determined by calculating the response entropy given by Eq. (19). To simulate dose-response curves under conditions which optimize information transfer for a given receptor, we optimized the response amplitude by constraining the Class II parameters according to Eq. (14) for binding modulators and determined the optimal [M_T_] and κ numerically for enzymatic modulators, respectively.

### Quantification of features used to characterize the response

The *EC*_*50*_, Hill coefficient *h* and the receptor occupancy at half maximum were determined as previously described [42]. In brief, the dose-response curves were normalized to the minimal and maximal output levels. *EC*_*50*_ values were determined from the signal concentration [S_T_] that results in 50 % of the normalized response. At this concentration the elasticity ε of the normalized curve was calculated numerically from Eq. (12). Finally to determine the receptor occupancy we determined the fraction of modulator proteins that is bound to the signal from the numerical simulations.

## Ethics approval and consent to participate

Not applicable.

## Consent for publication

Not applicable.

## Availability of data and material

All data supporting our findings is contained within this article.

## Additional file

Additional file 1: Derivations and additional analysis. This file contains derivations of Eq. (13)–Eq. (16) (DOCX 25 kb)

## Abbreviations

EC_50_: half maximal effective concentration
SAMP: switchable allosteric modulator protein
