# Supplementary material for: Molecular and cellular factors control signal transduction via switchable allosteric modulator proteins (SAMPs)
Source: BMC Syst Biol. 2016 Apr 27;10:35. doi: 10.1186/s12918-016-0274-3 (PMC4849100; doi:10.1186/s12918-016-0274-3)
Supplement: Additional file 1: — Derivations and additional analysis. This file contains derivations of Eq. (13)–Eq. (16) (DOCX 25 kb) [file 12918_2016_274_MOESM1_ESM.docx]

## Derivation and additional analysis

## S1 Response amplitude of a binding modulator system

To obtain the steady state solution for the unbound response regulator *R*, we set Eq.(1a-c) to zero and combine them with the mass conservation Eq. (3a-c) to obtain:

$K_{1}=\frac{(-[MR]+\left[ R \right]_{T}-[SMR])(-[MR]+\left[ M \right]_{T}-[SM]-[SMR])}{[MR]}$ (S 1a)

$K_{3}=\frac{(-[MR]+\left[ M \right]_{T}-[SM]-[SMR])(-[SM]-[SMR]+\left[ S \right]_{T})}{[SM]}$ (S 1b)

$K_{5}=\frac{[SM](-[MR]+\left[ R \right]_{T}-[SMR])}{[SMR]}$ (S 1c)

We next derived an expression for [*SM*] from equation (S1c) and two expressions for [*SMR*] from equations (S1a) and (S1b). Inserting [*SM*] into the equations for [*SMR*] resulting from (S1a) and (S 1b) yields two expressions for [*SMR*] as a function of [*MR*].

If the system is subjected to a saturating stimulus, i.e. $\left[ S \right]_{T}\to\infty$ we find that [*MR*] = 0. Under this condition, the steady state concentration of the regulator is given by:

$\left[ R \right]_{S,\infty}=\left[ R \right]_{T}- \frac{1}{2}(K_{5}+\left[ M \right]_{T}+\left[ R \right]_{T}-\sqrt{\left( \left[ R \right]_{T}+K_{5}+\left[ M \right]_{T} \right)^{2}-4\left[ M \right]_{T}\left[ R \right]_{T}})$ (S 2a)

On the other hand, the steady state concentration of the response regulator prior to stimulation is given by:

$\left[ R \right]_{0, \infty}=\left[ R \right]_{T}-\frac{1}{2}{(\left[ R \right]}_{T}+K_{1}+\left[ M \right]_{T}-\sqrt{\left( \left[ R \right]_{T}+K_{1}+\left[ M \right]_{T} \right)^{2}-4\left[ M \right]_{T}\left[ R \right]_{T}})$ (S 2b)

We thus obtain the response amplitude *A* given in Eq.(13) by subtracting Eq.(S2a) and Eq.(S2b) from each other and normalizing to [R_T_].

The optimal $\mu_{opt}$ (Eq.14) is obtained by setting the derivative of the response amplitude *A* Eq.(13) with respect to $\mu$ to zero and solving for $\mu$. Eq.(15) is obtained by replacing *µ* in Eq.(13) with Eq.(14).

## S2 Response amplitude of an enzymatic modulator system

We assume that the total concentration of signal exceeds the total concentration of regulator or modulator, i.e. that $\max\left( \left[ R \right]{}_{T},\left[ M \right]_{T} \right)\ll\left[ S \right]_{T}$. Hence in the mass conservations Eqs.(6) the concentrations $[SM]$ and $[SMR^{'}]$ can be neglected. Thus:

$\left[ S \right]_{T}\approx[S]$ (S 3)

Assuming that the complex formation reactions of the signal *S* with the modulator *M* or modulator regulator complex *MR’* are very fast, the complexes are in a quasi-steady state. Hence:

$\left[ SM \right]\approx\frac{\left[ S \right]\left[ M \right]}{K_{3}}$ (S 4a)

$\left[ SMR' \right]\approx\frac{\left[ S \right]\left[ MR' \right]}{K_{4}}$ (S 4b)

where, $K_{3}=k_{-3}/k_{3}$ and $K_{4}=k_{-4}/k_{4}$.

Eqs. (S 4) together with Eqs.(5) at steady-state yield the following relations:

$\left[ MR' \right]\approx\frac{\left[ M \right]\left[ R^{'} \right]}{K_{M}}$ (S 5a)

$\left[ SMR^{'} \right]\approx\frac{\left[ S \right]\left[ M \right]\left[ R^{'} \right]}{K_{3}K_{M}^{'}}$ (S 5b)

with $K_{M}=\frac{k_{2}+k_{-1}}{k_{1}}$ and $K_{M}^{'}=\frac{k_{6}+k_{-5}}{k_{5}}$:

Combining Eqs. (S 3) - (S 5), the mass conservations Eqs.(6) and the differential equation of the free regulator:

$\frac{d\left[ R \right]}{dt}=k_{2}\left[ MR^{'} \right]+k_{6}\left[ SMR^{'} \right]+\gamma\left[ R' \right]-\kappa[R]$ (S 6)

we can derive an expression for the concentration of the active regulator in the limit $\left[ S \right]_{T}\to\infty$:

$$\left[ R' \right]_{S,\infty}=-\frac{1}{2\left( \gamma+\kappa\right)}\left( k_{6}\left[ M \right]_{T}+K_{M}^{'}\left( \gamma+\kappa\right)+\kappa\left( \left[ M \right]_{T}-\left[ R \right]_{T} \right)-\sqrt{\left( 4 K_{M}^{'}\left[ R \right]_{T}\kappa\left( \gamma+\kappa\right)+\left( k_{6}\left[ M \right]_{T}+K_{M}^{'}\gamma+\left( K_{M}^{'}+\left[ M \right]_{T}-\left[ R \right]_{T} \right)\kappa\right)^{2} \right)} \right)$$

(S 7a)

Analogously to the binding modulator system, the enzymatic modulator system can be solved for [S_T_]=0:

$$\left[ R' \right]_{0,\infty}=-\frac{1}{2\left( \gamma+\kappa\right)}\left( k_{2}\left[ M \right]_{T}+K_{M}\left( \gamma+\kappa\right)+\kappa\left( \left[ M \right]_{T}-\left[ R \right]_{T} \right)-\sqrt{\left( 4 K_{M}\left[ R \right]_{T}\kappa\left( \gamma+\kappa\right)+\left( k_{2}\left[ M \right]_{T}+K_{M}\gamma+\left( K_{M}+\left[ M \right]_{T}-\left[ R \right]_{T} \right)\kappa\right)^{2} \right)} \right)$$

(S 7b)

We thus obtain the response amplitude given in Eq.(16) by subtracting Eq.(S7a) and Eq.(S7b) from each other and normalizing to [*R_T_*].

To optimize *µ*, we focused on an enzymatic modulator with *α_E_*=0. We thus set *α_E_* to zero in Eq.(16). and take the derivative with respect to *μ* and set it to zero. This yields Eq.(17).
